# Spectrum of Maternofetal Outcomes during Dengue Infection in Pregnancy: An Insight

**DOI:** 10.1155/2016/5046091

**Published:** 2016-03-16

**Authors:** Swati Sharma, Sandhya Jain, Shalini Rajaram

**Affiliations:** Department of Obstetrics and Gynaecology, University College of Medical Sciences and Guru Tegh Bahadur Hospital, New Delhi 110095, India

## Abstract

Dengue is a vector transmitted viral infection; tropical and subtropical countries see outbreaks of dengue each year. There is a paucity of literature on effects of dengue infection on pregnancy outcome and this prompted us to undertake a study for better understanding of pregnancy implications with dengue infection. Pregnant women admitted during the seasonal outbreak of dengue between September 2015 and October 2015 were studied and maternal and fetal outcomes in sixteen NS1Ag positive women were analysed. Out of sixteen women diagnosed with dengue fever, three had dengue shock syndrome (DSS) and eight had dengue haemorrhagic fever (DHF). The most common obstetric complication seen in 43% of the cases was oligohydramnios. Bleeding manifestations occurred in seven women and there were three maternal deaths. Perinatal complications included three intrauterine deaths, six nursery admissions, and one neonatal death. Thus dengue infection was associated with high maternal and perinatal mortality. In view of poor obstetric outcomes, this viral infection warrants early admission and prompt management.

## 1. Introduction

Dengue is a mosquito-borne viral disease. It is caused by viruses of the genus* Flavivirus*, family Flaviviridae, and Group IV ssRNA. Dengue is transmitted to humans by the mosquito* Aedes aegypti* and is one of the most rapidly spreading viral infections. There has been a 30-fold increase in its incidence in the last 50 years coupled to increasing migration from rural to urban areas. Annually 50 million people acquire dengue infection worldwide [1]. Dengue remains a major health concern for Southeast Asian countries with cyclic epidemics. In India multiple viral serotypes are circulating and some regions have case fatality rates of 3–5% in general population, which is much higher than other Southeast Asian regions (1%) [2].

Complications of dengue in pregnancy have been scarcely studied. Literature suggests an increased risk of maternal hemorrhage, preterm labour, and oligohydramnios [3–5]. Clinical presentation of dengue may be confused with HELLP syndrome, both conditions having elevated liver enzymes, haemolysis, and low platelet counts; however serology helps in distinguishing the two conditions. Low platelet counts pose a risk of postpartum hemorrhage. As reported in previous studies, dengue is associated with preterm births, low birth weight, fetal deaths, and vertical transmission leading to neonatal thrombocytopenia requiring platelet transfusions [6–10].

In a quest to answer vital issues we critically analysed dengue antigen positive cases in our institute and studied maternal and fetal outcomes.

## 2. Material and Method

This study was conducted in the Department of Obstetrics and Gynaecology of a tertiary level government hospital, Delhi, India. During the months of September and October 2015, 60 pregnant women were seen in the emergency room with complaint of fever and were admitted for evaluation. As per protocol, dengue PCR (NS1Ag) was done in all women. Out of 60, 16 were dengue positive.

Dengue was diagnosed using NS1Ag and/or IgM serology. Clinical grading was done according to WHO classification and case definitions. An acute febrile illness with two or more clinical manifestations like headache, retroorbital pain, myalgia, arthralgia, rash, hemorrhagic manifestation, or leukopenia and positive serology or occurrence at a time of dengue outbreak was taken as the definition of dengue fever (DF). Dengue hemorrhagic fever (DHF) was classified as fever, hemorrhagic tendencies, thrombocytopenia, evidence of plasma leakage, association of hepatomegaly, and circulatory disturbances. Increase in serial hematocrit was taken as evidence of plasma leakage and ultrasound showing pleural effusion, ascites, and gall bladder edema were taken as supportive evidence. Dengue shock syndrome (DSS) was classified when DHF symptoms included rapid and weak pulse, narrow pulse pressure of less than 20 mmHg, and hypotension [2].

Patients were managed with antipyretics, adequate hydration, and blood product transfusion as necessary. Strict maternal and fetal surveillance were done to identify complications early. Platelet counts were done two to three times per day depending on clinical profile. Obstetric data, clinical, laboratory parameters, and maternal and fetal outcomes of women are depicted in Table 1. The platelet count and hematocrit variation of the cases are shown in Table 2.

## 3. Results and Discussion

A majority of women were unbooked and presented with classical symptoms of high grade fever, myalgia, headache, and rashes to the emergency room. All women were young, age ranging from 22 to 32 years; mean age was 25 years. Out of 16 cases 13 presented in third trimester and two in second trimester and one was referred after LSCS was done in another hospital. She presented with large rectus sheath hematoma following caesarean section needing surgical exploration and drainage. None of the women had any underlying diseases or comorbidity except one (#case 5) who had moderate anemia on admission.

In the current study, eight women (50%) had dengue haemorrhagic fever (DHF). Ten women required transfusion to maintain platelet count in desired range; on average each woman required six platelet transfusions. Most women had low platelet counts at the time of admission (mean 81,000/mm^3^); 62% had platelets less than 50,000/mm^3^. Platelet count less than 30,000/mm^3^ was seen in dengue shock syndrome with increased risk of bleeding manifestations. A Sri Lankan case series reported 10 women with DHF but only eight required platelet transfusion [5]. Three women succumbed to dengue shock syndrome in current study with a mortality rate of 18% compared to 3–5% case fatality rate in rest of the India in nonpregnant population [2]. In another study done in India by Agrawal et al. maternal mortality rate was 12% [3].

Symptomatic dengue infection may increase the risk of preterm labour and, hence, low birth weight (LBW) as suggested by previous studies [3–5, 11]. In the study under discussion two women had preterm delivery (12%). Most women presented near term (*n* = 10) and of the four who presented in early gestation two had preterm labour. Preterm births reported were as low as 11% in the French Guiana study versus 41% in the study by Basurko et al. [4, 11]. One woman had spontaneous abortion (6%) and seven (43%) pregnancies were complicated with oligohydramnios in present study compared to 52% rate of oligohydramnios from a study in India by Agrawal et al. [3].

Bleeding manifestations were seen in seven women and three (19%) had postpartum hemorrhage (PPH) (#2, 11, and 13). One of them was diagnosed as DSS and the other two were diagnosed as DF. Both women diagnosed as DF had atonic PPH. One of them had PPH 48 hours following the delivery. Both the patients did not have any other bleeding manifestations and signs of plasma leakage hence were classified as only DF. In retrospective study by Basurko et al. on 53 pregnant women, PPH was reported in 10% of the cases compared to 19% in our study [4].

There were six neonatal intensive care unit (NICU) admissions (37%), three intrauterine deaths (IUD) (18%), and one neonatal death (6%). One neonatal death was caused by meconium aspiration syndrome. None of the neonates in our study showed symptoms of dengue such as thrombocytopenia, anemia, fever, or bleeding manifestations. Diagnostic tests for dengue were not routinely done for asymptomatic neonates. There are a number of studies on poor fetal outcome following dengue infection in parturient females. Vertical transmission of dengue has also been reported [4, 5]. Basurko et al. reported 5.3% vertical transmission rate in their study [4]. They also reported IUD rate of 3.8% and neonatal death rate of 1.9% while Carles et al. also reported high fetal death rate [4, 12].

Pregnant women with dengue fever should be considered for admission early because of its unpredictable course. Maintaining normothermia and adequate hydration should be the goal. Low platelet counts should be taken as a marker of severity of the disease. Oligohydramnios is an ominous sign when seen concomitantly with dengue infection because the high fetal mortality was associated with it. The exact cause of oligohydramnios is not known but dehydration associated with dengue fever may be contributory. Hydration should be maintained by encouraging oral intake of oral rehydration solution (ORS), fruit juice, and other fluids containing electrolytes and glucose for replacing losses from fever and associated vomiting [2]. Dengue fever does not warrant any active obstetrical intervention.

Another dilemma faced was in differentiating dengue infection from HELLP syndrome as both have somewhat similar laboratory parameters; however serology will help clinch the diagnosis. Careful examination and high index of suspicion by the treating obstetrician are required to diagnose and manage such cases especially during epidemics.

## 4. Conclusions

Our knowledge on effect of dengue on pregnancy is somewhat limited. In this study dengue was associated with high maternal and fetal morbidity and mortality. Dengue needs early diagnosis and prompt treatment to avoid its adverse outcomes. Pregnant women should avoid travel to dengue afflicted regions. Vector control methods should be employed during seasonal outbreaks. Awareness programs and medical education programs on the management of the dengue in pregnancy should be initiated especially during outbreaks to provide quality care.

## Figures and Tables

**Table 1:** Clinical profile of NS1Ag positive patients.

	Parity	POG weeks + days	IgM	Bleeding manifestations	Obstetrics complication	ICU admission	Maternal morbidity	Neonatal outcome
1	G_3_P_2_L_2_	39	+	N	Oligo, IUD	Y	Dengue induced hepatitis, pulmonary edema, and death	IUD

2	G_4_P_3_L_3_	38 + 4	−	Y (PPH)	Oligo, LSCS	Y	PPH, death	NICU (neonatal death-meconium aspiration syndrome with respiratory failure)

3	Primi	34 + 4	+	N	PTVD	Y	Preterm delivery, death	NICU (prematurity)

4	Primi	39	+	N	Oligo, NVD	N	None	NICU (respiratory distress)

5	G_2_P_1_L_1_	36 + 4	−	N	Oligo, NVD	N	None	Normal

6	G_3_P_2_L_2_	24 + 4	−	N	Oligo, NVD	N	None	Normal

7	G_3_P_2_L_2_	36	−	N	Oligo, FGR, NVD	N	None	NICU (LBW)

8	G_2_P_1_L_1_	38	+	N	Oligo, IUD	N	None	IUD

9	G_2_P_1_L_1_	17 + 4	−	Y (BPV)	Spontaneous abortion	N	Abortion	Abortion

10	P_2_L_2_	Post LSCS	−	Y (hematoma)	Rectus sheath hematoma	N	Hematoma drainage	Normal

11	G_3_P_2_L_1_	40	−	Y (2° PPH)	NVD	N	2° PPH	NICU (meconium aspiration)

12	Primi	34 + 2	−	Y (epistaxis)	NVD	N	Epistaxis	Normal

13	Primi	39	−	N	Forceps delivery	N	PPH	Normal

14	G_2_P_1_L_1_	32 + 2	+	Y	PTVD	N	Preterm delivery	NICU (prematurity)

15	G_3_P_2_L_2_	37 + 5	−	N (bleeding P/R, petechiae, and subconjunctival hemorrhage)	NVD	N	None	Normal

16	Primi	38 + 5	−	Y (hematuria)	IUD	N	None	IUD

G: gravidae, P: parity, L: living issue, VD: vaginal delivery, NVD: normal vaginal delivery, IUD: intrauterine death, PTVD: preterm vaginal delivery, PPH: postpartum hemorrhage, 2° PPH: secondary PPH, LBW: low birth weight, oligo: oligohydramnios, Y: yes, N: no, and NICU: neonatal intensive care unit.

**Table 2:** Hematological indices and their variation during hospital stay.

	Platelet count (10^3^/*µ*L)		Hematocrit (%)			Blood product transfusion	Diagnosis
	Admission	Least	Admission	Maximum	Least		
1	24	22	39	45	36	Y	DSS
2	18	18	33	37.6	33	Y	DSS
3	79	19	35	37	31.1	Y	DSS
4	123	123	29	29	28	N	DF
5	40	24	23.4	30	23.4	Y	DHF
6	45	45	30	31	28	N	DF
7	170	14	32.4	34.9	29	Y	DHF
8	24	20	27.8	30	27	Y	DHF
9	18	18	42	42	31	Y	DHF
10	32	32	38	44	33	Y	DHF
11	90	62	33	36	33	N	DF
12	190	43	32	39	32	N	DHF
13	76	76	29	33	29	N	DF
14	42	39	40	41	35	Y	DHF
15	310	160	31	32	32	N	DF
16	16	16	39	39	30	Y	DHF

## References

[1] Rigau-Pérez J. G., Clark G. G., Gubler D. J., Reiter P., Sanders E. J., Vorndam A. V. (1998). Dengue and dengue haemorrhagic fever. *The Lancet*.

[2] (2009). *WHO Dengue: Guidelines for Diagnostics, Treatment, Prevention and Control*.

[3] Agrawal P., Garg R., Srivastava S., Verma U., Rani R. (2014). Obstetrics and gynecology pregnancy outcome in women with Dengue infection in northern India. *Indian Journal of Clinical Practice*.

[4] Basurko C., Carles G., Youssef M., Guindi W. E. L. (2009). Maternal and foetal consequences of dengue fever during pregnancy. *European Journal of Obstetrics Gynecology & Reproductive Biology*.

[5] Kariyawasam S., Senanayake H. (2010). Dengue infections during pregnancy: case series from a tertiary care hospital in Sri Lanka. *Journal of Infection in Developing Countries*.

[6] Dale Carroll I., Toovey S., Gompel A. V. (2007). Dengue fever and pregnancy—a review and comment. *Travel Medicine and Infectious Disease*.

[7] Chotigeat U., Kalayanarooj S., Nisalak A. (2003). Vertical transmission of dengue infection in thai infants: two case reports. *Journal of the Medical Association of Thailand*.

[8] Chye J. K., Lim C. T., Ng K. B., Lim J. M. H., George R., Lam S. K. (1997). Vertical transmission of dengue. *Clinical Infectious Diseases*.

[9] Maroun S. L. C., Marliere R. C. C., Barcellus R. C., Barbosa C. N., Ramos J. R. M., Moreira M. E. L. (2008). Case report: vertical dengue infection. *Jornal de Pediatria*.

[10] Fatimil L. E., Mollah A. H., Ahmed S., Rahman M. (2003). Vertical transmission of dengue: first case report from Bangladesh. *The Southeast Asian Journal of Tropical Medicine and Public Health*.

[11] Friedman E. E., Dallah F., Harville E. W. (2014). Symptomatic dengue infection during pregnancy and infant outcomes: a retrospective cohort study. *PLoS Neglected Tropical Diseases*.

[12] Carles G., Talarmin A., Peneau C., Bertsch M. (2000). Dengue fever and pregnancy. A study of 38 cases in French Guiana. *Journal de Gynécologie, Obstétrique et Biologie de la Reproduction (Paris)*.

